# Effects of general and corona-specific stressors on mental burden during the SARS-CoV-2 pandemic in Germany

**DOI:** 10.3389/fpubh.2022.991292

**Published:** 2022-11-22

**Authors:** Lara Hubenschmid, Isabella Helmreich, Göran Köber, Donya Gilan, Svenja B. Frenzel, Rolf van Dick, Klaus Lieb

**Affiliations:** ^1^Leibniz Institute for Resilience Research (LIR), Mainz, Germany; ^2^Institute of Medical Biometry and Statistics, Faculty of Medicine and Medical Center – University of Freiburg, Freiburg im Breisgau, Germany; ^3^Freiburg Center for Data Analysis and Modeling, University of Freiburg, Freiburg im Breisgau, Germany; ^4^Department of Psychiatry and Psychotherapy, University Medical Center of the Johannes Gutenberg University Mainz, Mainz, Germany; ^5^Department of Psychology, Goethe-University Frankfurt, Frankfurt, Germany

**Keywords:** resilience, mental burden, risk factors, protective factors, vulnerable groups, SARS-CoV-2, stressor loads

## Abstract

The SARS-CoV-2 pandemic turned out to be a serious threat to mental and physical health. However, the relative contribution of corona-specific (DH_s_) and general stressors (DH_g_) on mental burden, and specific protective and risk factors for mental health are still not well understood. In a representative sample (*N* = 3,055) of the German adult population, mental health, potential risk, and protective factors as well as DH_s_ and DH_g_ exposure were assessed online during the SARS-CoV-2 pandemic (June and July 2020). The impact of these factors on mental health was analyzed using descriptive statistics, data visualizations, multiple regressions, and moderation analyses. The most burdensome DH_g_ were financial and sleeping problems, respectively, and DH_s_ corona-media reports and exclusion from recreational activities/important social events. 31 and 24% of total mental health was explained by DH_g_ and DH_s_, respectively. Both predictors combined explained 36%, resulting in an increase in variance due to DH_s_ of only 5% (R^2^ adjusted). Being female, older and a lower educational level were identified as general risk factors, somatic diseases as a corona-specific risk factor, and self-efficacy and locus of control (LOC) proved to be corona-specific protective factors. Further analyses showed that older age and being diagnosed with a somatic illness attenuated the positive influence of LOC, self-efficacy, and social support on resilience. Although the data showed that after the first easing restrictions, the stressor load was comparable to pre-pandemic data (with DH_s_ not making a significant contribution), different risk and protective factors could be identified for general and corona-specific stressors. In line with observations from network analysis from other groups, the positive impact of resilience factors was especially diminished in the most vulnerable groups (elderly and somatically ill). This highlights the need to especially target these vulnerable groups to foster their resilience in upcoming waves of the corona pandemic.

## Introduction

The SARS-CoV-2 (severe acute respiratory syndrome coronavirus 2) pandemic has shown to be a threat to physical as well as mental health [e.g., (1, 2)]. Not only the existential fears evoked by the virus itself (fear of illness, suffering, and death) are stressful, but also the regulatory policies such as lockdown and their resulting social distancing, self-isolation, financial insecurity, or travel restrictions. Moreover, secondary consequences such as an impending economic crisis and recession are feared (3, 4). Different systematic reviews showed that the COVID-19 pandemic evoked significant increases in depression, anxiety, and posttraumatic stress symptoms in the general population during (5, 6) and after the first (3, 7) and subsequent COVID waves (8, 9). In the general population, studies also showed slightly higher levels of psychological distress compared to pre-pandemic data (3, 10, 11). Quarantine measures appeared to have a particularly negative impact on psychological wellbeing, with higher prevalence rates of psychological distress symptoms (e.g., irritability, insomnia, and emotional exhaustion) and mental disorders (e.g., depression) demonstrated thereafter [e.g., (12, 13)]. Both, fear appeals, that have intentionally been used to increase compliance rates for infection control measures (14, 15), as well as the spread of rumors (16) have also been shown to have negative psychological effects (including the loss of trust in mental health services or policies) (13).

Thereby, those with chronical illness or poorer health (7, 9, 17) or a relative or acquaintance infected with COVID-19 (17, 18) as well as women (3, 11, 19–23) were identified as particularly vulnerable. Further risk factors were catastrophizing thinking, the personality trait neuroticism, and the need for instrumental support (2). Research shows that specific resilience factors (as social support or optimism) help humans to cope with stressful life events (24, 25) and mitigate risk factors (26–28). There is corresponding evidence that some of those factors, e.g., positive appraisal and optimism (2, 29), perceived social support (18, 30, 31), self-efficacy, cognitive flexibility (32) and locus of control (LOC) (7, 30, 33), strengthened mental health during the pandemic. It should be noted, however, that these strategies were more difficult to apply (e.g., optimism) during periods of closure or constant negative news (34).

Although corona-specific stressors significantly contribute to the total stressor load experienced during a pandemic, also general micro-stressors (35, 36) or “daily hassles” [“demands that, to some degree, characterize everyday transactions with the environment and are classified as irritating, frustrating, and unsettling” (37), p. 3] are present, which also contribute to the total stressor load. Daily hassles are considered a good predictor of psychological symptoms, because they involve immediate adjustment processes (37) and may also have profound negative effects on mental health, especially if they are numerous and enduring (38). Seery et al. (39) examined u-shaped relationships across the lifespan between adversities and wellbeing (lower global distress, posttraumatic stress symptoms and functional impairment, and higher life satisfaction). Their results showed that individuals who experienced less lifetime adversity suffered comparatively more from being confronted with current adversity compared to people with moderate lifetime adversities. However, when there were too many cumulative stressors, this turned into a negative effect on wellbeing (e.g., stress-associated diseases).

### Objective

**Figure 1:**
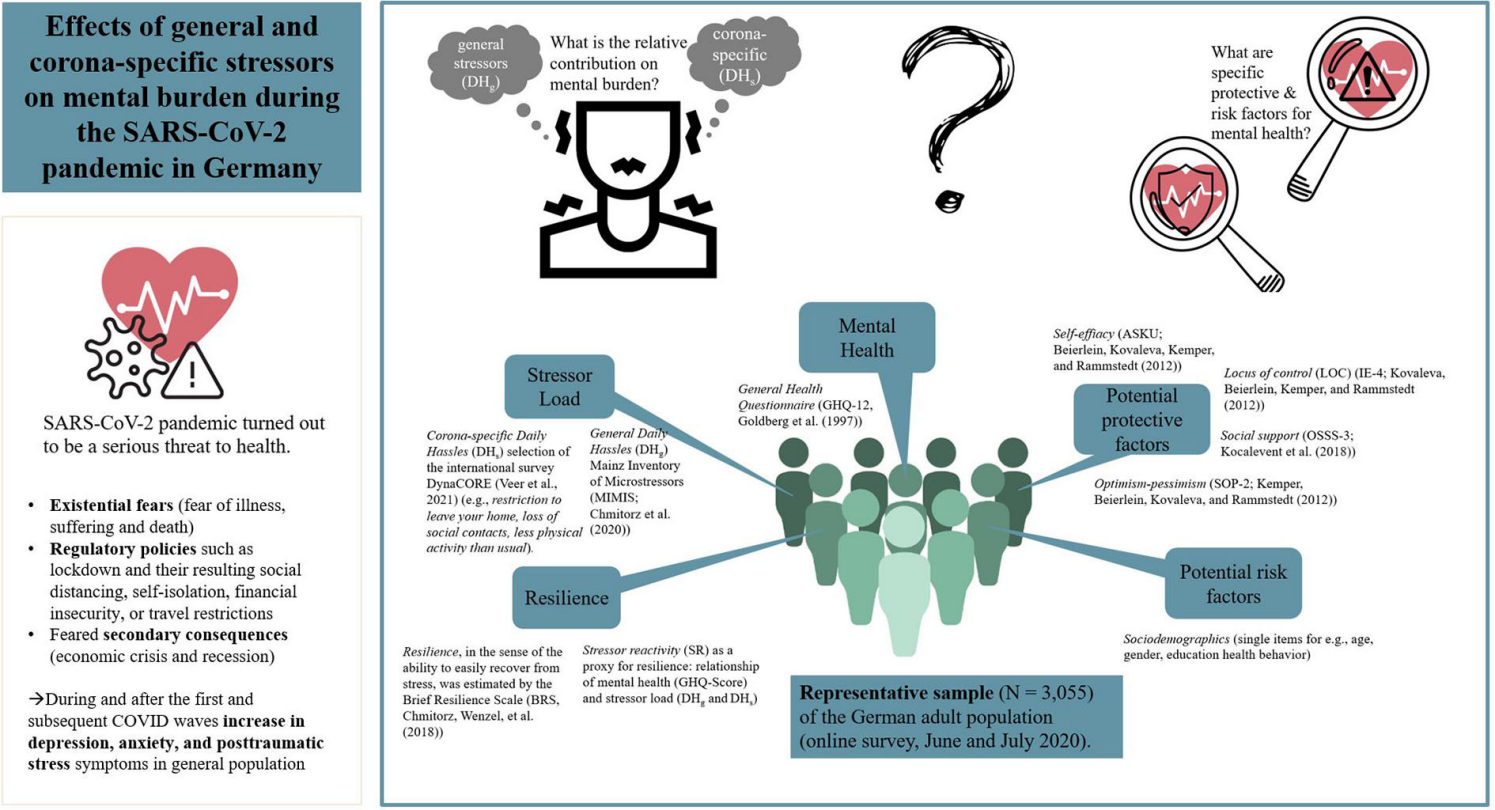
Graphical representation of the study aims.

Although numerous studies have been published researching stressor load as well as risk and resilience factors during the pandemic, only a few examined this in representative samples (3). Moreover, none of these studies examined the specific contribution of corona-specific (DH_s_) and general stressors (DH_g_) and their relative impact on mental burden during the pandemic. Our study, therefore, used a representative sample of the German population in order to investigate: (1) the stressor load (DH_s_ and DH_g_ and their relative contributions) in the German population during the SARS-CoV-2 pandemic in summer 2020, (2) the relative impact of DH_s_ and DH_g_ and their combination on mental health, and (3) risk and resilience factors with a special focus on their relevance in vulnerable groups such as older people. The study aim is also graphically represented in Figure 1.

## Materials and methods

### Sample

A representative sample (*N* = 3,055) of the adult German population (18+ years) was assessed in cooperation with the infratest dimap polling institute between June 26th and July 19th, 2020. This time period was characterized by a steady decline in 7-day incidence (3.3 per 100,000 cases) after the first wave of the pandemic in March 2020. The hospitalization rate was 17%, and the mortality rate was just under 5%. Testing capacity was significantly expanded, and the Corona alert app for contact tracking was just introduced (40). Contact restrictions were lifted, stores and restaurants reopened, and schools resumed regular operations. Nevertheless corona-specific protective measures and restrictions (e.g., social distancing) and fears of infection and of the next wave were present (41). By using a weighting variable (with the variables age, education, state, and gender), slightly overrepresented populations can be weighted downward, and underrepresented populations can be weighted upward in the dataset so that the weighted dataset reflects the population. All calculations were computed once with and once without the weighting variable, and no differences emerged. We adopted the minimal correction by using the weighting variable. The design weights were used throughout the manuscript. The sample characteristics are shown in Table 1.

**Table 1:** Demographic characteristics of the sample.

	**Overall (*N* = 3,055)**	**Males (*n* = 1,494)**	**Females (*n* = 1,561)**
**Age (years)**			
Mean (*SD*)	50.59 (*17.25*)	49.40 (*17.49*)	51.06 (*17.67*)
Median (*Min; Max*)	52.00 (*18;93*)	50.00 (*18;93*)	52.00 (*18;91*)
**Education (frequency**, ***%*****)**			
No school-leaving qualification	40 (*1.3%*)	17 (*1.2%*)	23 (*1.5%*)
Still in school education	15 (*0.5%*)	5 (*0.4%*)	9 (*0.6%*)
Low secondary education	1,013 (*33.1%*)	493 (*33.6%*)	520 (*33.3%*)
Medium secondary education	964 (*31.5%*)	438 (*29.3%*)	526 (*33.7%*)
High school	470 (*15.4%)*	233 (*15.6%)*	236 (*15.1%*)
University degree	555 (*18.2%)*	309 (*20.7%)*	245 (*15.7%*)
**Somatic illness (frequency, %)**			
Self reported diagnosis	1,231 (*40.3%*)	554 (*37.00*%)	676 (*43.4*%)
no diagnosis	1,812 (*59.3*%)	939 (*62.7*%)	873 (*56.1*%)
N/A	12 (*0.4%*)	5 (*0.3*%)	7 (*0.4*%)
**Mental disorder (frequency, %)**			
Self reported diagnosis	350 (*11.5*%)	136 (*9.1%*)	213 (*13.7%*)
no diagnosis	2,680 (*87.7%*)	1,353 (*90.3%*)	1,327 (*85.3%*)
N/A	25 (*0.8%*)	9 (*0.6%*)	16 (*1%*)
**Self estimated resilience (BRS)**			
Mean (*SD*)	3.31 (*0.78*)	3.41 (*0.74*)	3.22 (*0.79*)
Median (*Min, Max*)	3.34 (*1;5*)	3.33 (*1;5*)	3.17 (*1;5*)
**Mental health (GHQ-12)**			
Mean (*SD*)	12.49 (*6.58*)	11.71 (*6.23*)	13.25 (*6.81*)
Median (*Min; Max*)	11 (0*;36*)	10 (0*;36*)	12 (0*;36*)
**DH**_**s**_ **(burden)**			
Mean (SD)	1.82 (*0.91*)	1.80 (*0.91*)	1.85 (*0.91*)
Median (Min; Max)	1.77 (*0;5*)	1.70 (*0;5)*	1.77 (*0;5)*
**DH**_**s**_ **(binary count)**			
Mean (SD)	9.54 (*3.54*)	9.67 (*3.53)*	9.46 (*3.54*)
Median (Min; Max)	11 (*0;14*)	11 (*0;14*)	11 (*0;14*)
**DH**_**g**_ **(burden) (MIMIs)**			
Mean (SD)	1.20 (*0.81*)	1.24 (*0.81*)	1.17 (*0.82*)
Median (Min; Max)	1.10 (*0;3.09*)	1.18 (*0;2.97*)	1.03 (*0;3.09*)
**DH**_**g**_ **(frequency) (MIMIS)**			
Mean (SD)	68.22 (*47.60*)	67.56 (*46.84*)	68.85 (*48.33*)
Median (Min; Max)	58.00 (*0;310*)	58.00 (*0;294*)	58.00 (*0;310*)
**Optimism (SOP-2)**			
Mean (*SD*)	5.02 (*1.46*)	5.03 (*1.42*)	5.01 (*1.49*)
Median (*Min; Max*)	5.50 (*1;7*)	5.5 (*1;7*)	5.5 (*1;7*)
**Self-efficacy (ASKU-4)**			
Mean (*SD*)	3.89 (*0.73*)	3.93 (*0.71*)	3.86 *(0.75*)
Median (*Min; Max*)	4.00 (*1;5*)	4.00 (*3;5*)	4.00 (*1;5*)
**Social support (OSSS-3)**			
Mean (*SD*)	9.81 (*0.73*)	9.69 (*1.92*)	9.92 (*2.07*)
Median (*Min; Max*)	10.00 (*3;14*)	10.00 (*3;14*)	10.00 (*3;14*)

*N*, number of population; *n*, number of cases; SD, Standard Deviation; Min, minimum; Max, maximum; N/A, not applicable; BRS, Brief Resilience Scale; GHQ-12, General Health Questionnaire (12 item version); DH_g_, general daily hassles; DH_s_, corona-specific daily hassles; MIMIS, Mainz Inventory of Microstressors; SOP-2, Skala Optimismus-Pessimismus-2; ASKU-4, Allgemeine Selbstwirksamkeit Kurzskala; OSSS-3, Oslo Social Support Scale-3.

### Survey questionnaire

The survey questionnaire contained 182 items and included the following sections: sociodemographics, mental health, resilience and resilience-associated constructs, stressor exposure as well as individual and social values. Except for the sociodemographic assessments, only validated questionnaires were used.

*Sociodemographics* were assessed using single items for, e.g., age, gender, or education as well as health behavior (in total 32 items). Based on the variables education, occupation and income, a socioeconomic status (SES) index was created using a predefined scoring system (42) and from there dividing participants into five equally populated groups (quintiles). This resulted in a classification into low (1st quintile), medium (2nd−4th quintile) and high (5th quintile) SES. *Mental health* was assessed by the General Health Questionnaire [GHQ-12, Goldberg et al. (43), 12 items on a 4-point Likert scale] for the last few weeks. *Resilience*, in the sense of the ability to easily recover from stress, was estimated by the Brief Resilience Scale [BRS, Chmitorz et al. (44); 6 items on a five-point Likert scale—“strongly disagree” to “strongly agree”].Total scores were obtained by taking the mean of the item scores. Due to restrictions of the questionnaire length, only four well established *resilience-associated constructs* (45–47) were included: *optimism-pessimism* [SOP-2; (48), 2 items; from “not optimistic/pessimistic at all” to “very optimistic/pessimistic” on a seven-point Likert scale], *locus of control* (LOC) [IE-4; (49), 4 items; from “does not apply at all” to “applies fully” on a five-point Likert scale], *self-effiacy* [ASKU; (50), 3 items; from “does not apply at all” to “applies fully” on a five-point Likert scale], and *social support* [OSSS-3; (51), 3 items; response format differs on a 4 resp. 5-point Likert scale].

For *stressor exposure*, we assessed three different types of stressors, *general daily hassles* (DH_g_), *corona specific daily hassles* (DH_s_) and *life events* (LE). For assessing DH_g_, we used the Mainz Inventory of Microstressors [MIMIS; (52); 58 items]. The MIMIS assessed the frequency each stressor occurs (up to the last 7 days, DH_fg_) as well as the perceived burden (from “not at all burdensome” to “very burdensome” on a five-point Likert scale, DH_bg_). DH_s_ were measured with a selection of 13 items of the international survey DynaCORE (29), which had been introduced to assess stress due to the pandemic and respective measures (e.g., *restriction to leave your home, loss of social contacts, less physical activity than usual*). We asked whether a stressor occurred (on up to the last 7 days, total binary frequency, DH_fs_), and if so—how the burden was classified (from “not at all burdensome” to “very burdensome” on a five-point Likert scale, DH_bs_). For LE as macro stressors, we used a self-developed question that queried one LE within the last 12 months. The *Response to Stressful Experiences Scale* [RSES-4; (53), 4 items, “strongly disagree” to “strongly agree” on a five-point Likert scale] was used to determine the burden of it. Additionally, we used the Perceived Stress Scale [PSS-4; (54), 4 items “never” to “very often” on a five-point Likert scale] in order to assess the subjectively perceived stress. We also collected *Social Identification with social groups* [adapted from Doosje et al. (55), 4 items, “strongly disagree” to “strongly agree” on a five-point Likert scale], *values* [Individuelle reflexive Werte: (56); 16 items, “very unimportant” to “very important” on a seven-point Likert scale], *political attitudes* [Sozio-Politische Einstellungen, (57), 16 items, “correct” and “not correct”] and *cultural diversity* [Pro-diversity beliefs; adapted from Kauff and Wagner (58), 2 items, “not at all” to “full” on a four-point Likert scale]. The complete questionnaire is shown in Supplementary material 1. In this manuscript, we focus on the following outcomes and risk/protective factors: resilience (BRS), mental health (GHQ-12), stressor exposure (DH_g_, DH_s_) as well as risk (sociodemographic and health behavior) and protective factors (SOP-2, IE-4, ASKU, OSSS-3). Detailed results of the other items will be published elsewhere.

### Statistical analyses

For all statistical analyses a significance level of α = 0.05, two-tailed, was adopted. All analyses were conducted with the weighting variable included. Data analysis was performed in R (v4.2.0, www.r-project.org/), in particular the packages ggplot2 (59), effects (60) and lavaan (all regressions were calculated with the scores of the confirmatory factor analysis) (61). All analyses are exploratory in nature; hence, *p*-values and 95% confidence bands are descriptive and not corrected for multiple comparisons.

#### Stressor load

To investigate the most frequent and most burdensome DH, descriptive methods (mean values and frequencies) were used. In addition, (weighted) heat maps—frequency of general DH (DH_fg_) and burden of corona-specific DH (DH_bs_)—were generated. A clustered heat map is a data visualization technique for showing patterns based on color intensities. To obtain information about which stressors (general vs. corona-specific) had a stronger impact on mental health, we calculated a regression analysis with either DH_fg_, DH_fs_ or both combined (DH_fc_) as well as DH_bg_ and DH_bs_ or both combined (DH_bc_) (each modeled as a second-degree polynomial) as independent variable.

#### Operationalization of stressor reactivity as a proxy for resilience

Following Kalisch et al. (62), resilience has been defined as a mental health outcome. However, since this study was a cross-sectional study which did not allow for a longitudinal assessment of mental health outcomes, we used the relationship of mental health (here the GHQ score) and stressor load (general daily hassles and corona-specific daily hassles) as a proxy for resilience. The GHQ-DH regression curve shows the normative predicted stressor reactivity (SR). Subjects residuals, which deviate from the normative predicted SR, contain information about their individuals SR (i.e., their vulnerability/resilience level): If the individual residual is located above this curve, it expresses a relative over-reactivity (=vulnerable), a value below the curve expresses a relative under-reactivity (=resilience) (29, 45, 62). We aimed to assess the stressor load as objectively as possible and therefore summed up the frequency of occurrence of each stressor as a total sum value: DH_fg_ were counted continuously (Range: 0–406), DH_fs_ were counted binarily (Range: 0–13). For the conceptualization of resilience, three different univariate regression analyses were applied considering the best model fit to get the subjects' stressor reactivity (SR) score: The GHQ score served in each calculation as criterion and either DH_fg_, DH_fs_ or both combined (DH_fc_) as predictors. For convenience of interpretation, we calculated individuals' inverse residuals, so that high values indicate high resilience (63). The retrospectively assessed single, most burdensome life event didn't explain any variance in regression analysis, therefore, we omitted it from further analysis. The BRS, which measures the ability to recover from stress, is also frequently used as a resilience measure (44). It therefore served as a benchmark to compare the results of this study with those before and during the pandemic.

#### Vulnerable groups, protective factors, and in-depth analysis of previously identified vulnerable groups

To identify risk factors, we used moderation analyses with the GHQ score as criterion and DH_fg_ or DH_fs_ as predictor. Sociodemographic characteristics (age, gender, education) and health status (physical illness or mental disorder) were used as moderators of this analysis. We controlled either for DH_fg_ or DH_fs_. To identify protective factors, we also calculated the same moderation analyses, but with the different resilience factors (RF), i.e., optimism, self-efficacy, LOC and social support, as moderators. We controlled either for DH_fg_ and DH_fs_ and for age, gender, education, somatic illness, and mental disorder. To identify protective factors for the previously identified vulnerable groups, moderation analyses were calculated using SR score as the criterion and the RF as independent variables and the previously identified risk factors as moderators.

## Results

### Sample

The final sample included 3,055 participants. Table 1 shows the demographic characteristics. Around half of the participants were women (51.1%). Age was distributed equally between18 and 93 years (median age = 52.00 years; mean age = 50.59 years; SD = 17.25 years). About one-third had either the low secondary education (33.5%) or medium secondary education (33.1%), and nearly one-sixth of participants had a university degree (18.2%) or high school (15.4%). Only 1.3% of the participants had no school-leaving qualification or were still in school (0.5%). About 40% of the respondents reported to be affected by any somatic disease, about 12% by a mental disorder. The proportion of women was higher in both groups (43% in females vs. 37% in males and 14% in females and 9% in males, respectively). The mean BRS score of respondents was 3.31 (SD = 0.78), with men's scores (mean = 3.41, SD = 0.74) indicating higher self-estimated resilience than women's (mean = 3.22, SD = 0.74). Similar results were found regarding GHQ (mean = 12.49; SD = 6.58; lower values indicate better mental health): mean values were higher in women (mean = 13.25; SD= 6.81) than in men (mean = 11.71; SD = 6.23). The values of frequency and burden of DH and the resilience-associated constructs (e.g., optimism) are shown in Table 1 (total and gender-separated).

### Stressor load

**Figure 2:**
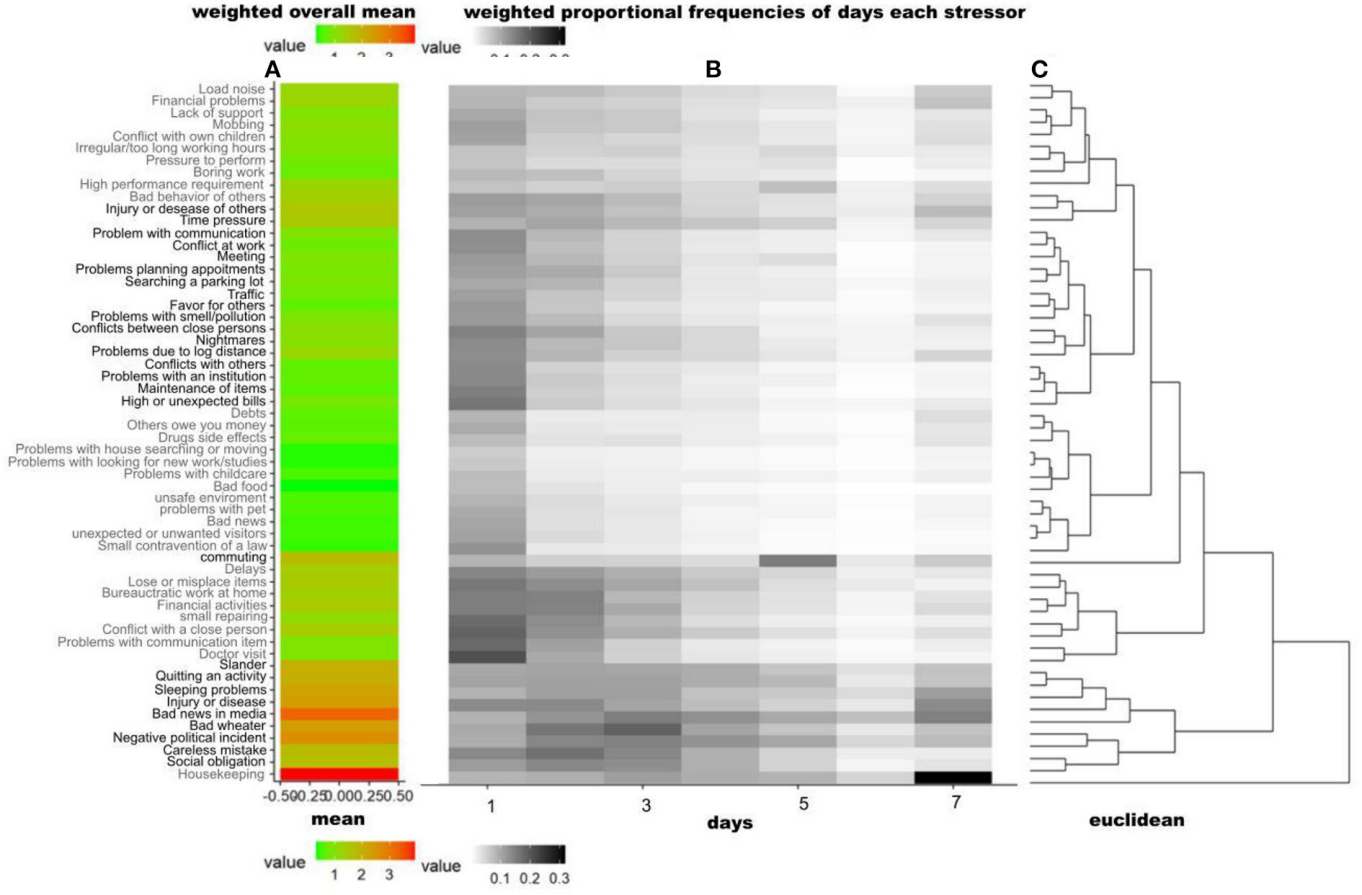
DH_fg_ Heat Map, indicating the overall and temporal frequencies of stressors (weighted DH_fg_) vertically clustered by their temporal occurrence patterns. The figure consists of three parts: **(A)** showing the weighted overall mean of each item. **(B)** showing the weighted proportional frequencies of days each stressor was experienced–the darker the background, the more people experienced the stressor on the corresponding number of days (1–7): for example, “housekeeping” were experienced by many respondents on 7 days, “Doctor visit” was also reported to have been experienced once that week or some individuals reported “commuting” on 5 days. **(C)** Dendrogram indicating the difference between the items and their clusters of temporal patterns of relative frequencies. This cluster solution was also used to vertically sort the stressor items (using hierarchical clustering, euclidean distance and complete linkage). The proportional frequencies of each item (e.g., housekeeping 1 day: 10%, 2 days 13%, …, 7 days 30%) are successively merged into clusters. The fusion of different clusters is marked in the dendrogram by vertical lines. The heterogeneity within the clusters is plotted on the x-axis, it is growing with increasing cluster size. Longer horizontal lines indicate an increase in heterogeneity (of clusters) between the temporal occurrence patterns of the items: **Cluster A** includes 10 items (“loud noise” to “bad behavior of others”). **Cluster B** includes 17 items (“injury or disease of others” to “maintenance of items”). **Cluster C** includes 12 items (“Depts” to “small contravention of a law”). **Cluster D** includes 8 items (“Delays” to “Doctor visit”). **Cluster E** includes “commuting”. **Cluster F** includes 9 items (“slander” to “social obligation”), and **Cluster G** includes “housekeeping”.

Our first aim was to gain insights into the stressor load of the German population during the SARS-CoV-2 pandemic: The most frequent and most burdensome DH_g_ and DH_s_ were examined. The most frequently reported DH_fg_ were housekeeping [reported by 83.64% of participants occurring on at least one and up to 7 day(s) in each case], followed by bad news in media (80.73%), negative political incident (72.42%), own physical complaints (65.95%), bad weather (71.15%), and sleeping problems (59.41%). Financial problems (average severity rating mean 2.31, possible answer range 1–4, percentage of those affected 28.5%), followed by sleeping problems (2.29; 51.30%), own physical complaints (2.13; 57.34%), and bad behavior of others and time pressure (both 2.10; 38.04% resp. 38.27%) were the most burdensome DH_bg_. Among the most frequently stated DH_g_, the average perceived burden was relatively low: housekeeping (1.23; 53.49%), negative political incident (1.71; 58.23%), and bad weather (1.61; 52.64%). To gain more knowledge about the temporal structure of the stressors, clustered heat maps for the DH_fg_ are presented in Figure 2: It shows the overall mean and temporal frequency patterns of stressors (weighted, DH_g_), which were vertically sorted according to the cluster solution. By visual inspection of the dendrogram, seven distinct clusters can be identified regarding the temporal occurrence: In Cluster A it is characteristic that DH_g_ tended to occur not at all or very often, i.e., on no or up to 7 days. In Cluster B DH_fg_ occurred rather rarely (i.e., once, or twice or three times). This temporal clustering also unveils some contentual structure: Cluster A and B primarily contain problems with others or at work (e.g., “boring work” or “problems with an institution”). Cluster C primarily concerns insecurity (related to financial, health, or environmental status). This cluster was characterized by a low stressor load, i.e., DH did not occur just once. Cluster D includes external conditions (e.g., “delays” to “doctor visit”). In Cluster D stressors load is slightly elevated, by stressor occurrence on either 1–2 days or not at all. Cluster E includes “commuting” and has a clear peak at 5 days (every workday). Cluster F primarily indicates internal problems (e.g., “slander” or “careless mistake”) and the frequency of occurrence is highest for this cluster (on at least one or two up to 7 days). And Cluster G (“housekeeping”), has the highest frequency of occurrence on 7 days.

**Figure 3:**
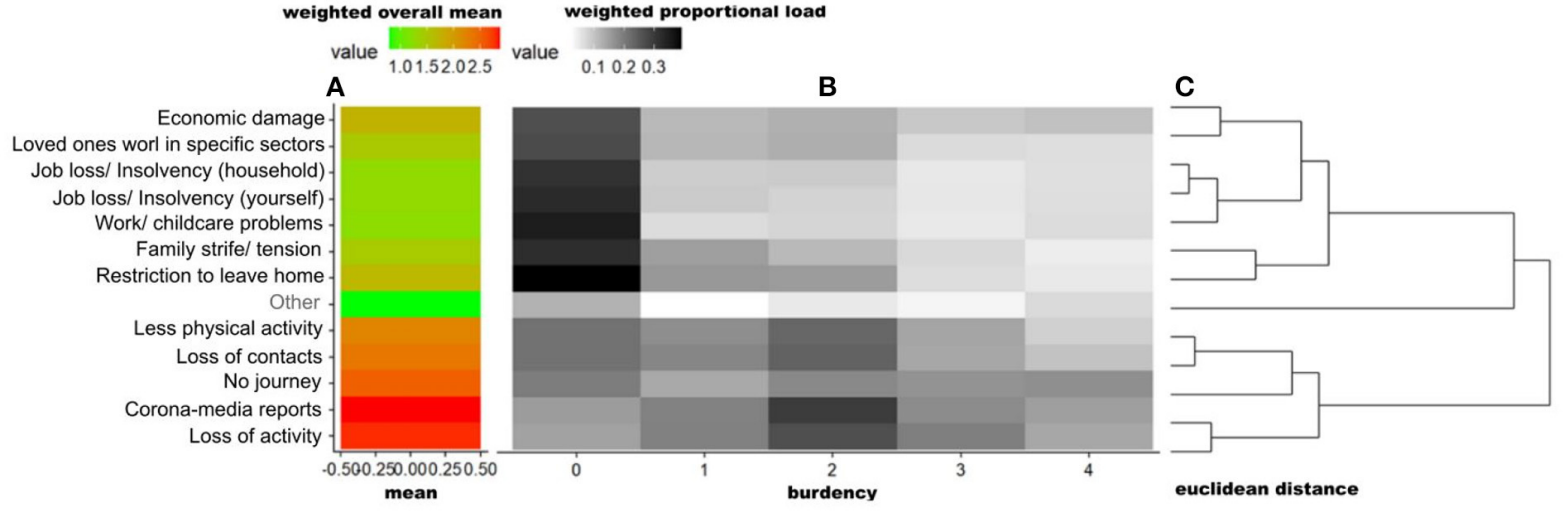
DH_bs_ Heat Map, indicating the burden's mean of stressors (weighted DH_bs_) vertically clustered by their burden patterns. The figure consists of three parts: **(A)** showing the weighted overall mean of each item. **(B)** illustrates the weighted proportional load with which a particular stressor was experienced–the darker the background, the more burdensome the stressor was experienced (from “not at all burdensome” to “very burdensome” on a five-point Likert scale). For example, “media-corona reports” were experienced by many respondents as burdensome (average severity rating mean: 2.88). **(C)** Dendrogram indicating the difference between the items and their clusters of patterns of relative burden. This cluster solution was also used to vertically sort the stressor items (using hierarchical clustering, euclidean distance and complete linkage). The proportional burden of each item (e.g., loss of activity “not at all burdensome”: 13%, …, “very burdensome” 10%) are successively merged into clusters. The fusion of different clusters is marked in the dendrogram by vertical lines. The heterogeneity within the clusters is plotted on the x-axis, it is growing with increasing cluster size. Longer horizontal lines indicate an increase in heterogeneity (of clusters) between the temporal occurrence patterns of the items: **Cluster A** includes 7 items (“economic damage” to “restriction to leave home”). **Cluster B** includes “Other” with answers concerning additional problems (“Goods & services problems”, “Burden due to mandatory masks”, “Deterioration of mental situation” or “Digital study/school”), fears (“Second wave”, “economic impact/lockdown”, “Social change”, “Infection itself/others” and “Further measures”) and anger (“Ignoring measures/recklessness”, “Measures/policy” and “Media reporting”). **Cluster C** includes 5 items (“Less physical activity” to “loss of activities”).

The most frequently reported corona-specific DH_fs_ were corona-media reports (reported by 96.60% of participants occurring on up to the last 7 days), followed by the loss of ability to participate in recreational activities or in important social events (93.29%), loss of social contacts (87.24%), less physical activity (83.84%), restrictions to leave home (81.87%), and economic damage (69.68%). The DH_bs_, sorted according to their degree of burden, are shown in the heat map of Figure 3. As shown in Figure 2A, which demonstrates the weighted overall mean of each stressor item, the most burdensome DH_s_ were corona-media reports (average severity rating mean: 2.88, possible answer range: 1–5, percentage of those affected: 96.60%), followed by the loss of ability to participate in recreational activities or in important social events (“Loss of activity”: 2.80; 93.52%), private or professional travel not feasible (“No journey”: 2.53; 85.07%), loss of social contacts (“Loss of contacts”: 2.35; 87.36%), and less physical activity than usual (“Less physical activity”: 2.23; 84.08%). As shown in Figure 3B, which illustrates the weighted proportional load with which a particular stressor was experienced, corona-specific stressors were clustered into 3 clusters according to their relative degree of stress and sorted vertically (see right part C). It was frequently stated that Cluster A's items, e.g., “Restriction to leave home” or “Work/ childcare problems”, occurred, but most of participants did not associate burden with them. Cluster B includes “Other”, which included answers concerning additional problems (e.g., “Goods and services problems”), fears (e.g., second wave) and anger (e.g., ignoring measures/recklessness). Cluster C, e.g., “corona-media reports”, most often had a medium load (2).

In order to analyze the perceived stressor load, we used univariate regression models: 31 and 24% of the variance in mental health outcome (GHQ score, dependent variable) was explained by DH_bg_ [R^2^ adjusted: 0.31; *F*_2,3054)_ = 683.74, *p* < 0.001] and DH_bs_ [R^2^ adjusted: 0.24; *F*_2,3054)_ = 481.77, *p* < 0.001], respectively. DH_bg_ and DH_bs_ were modeled as second-degree polynomial considering best model fit. In the model with combined stressors (DH_bc_), DH_bs_ explained only an additional 5% in the total perceived stressor load [determined by univariate regression with GHQ score as the dependent variable and DH_bg_ and DH_bs_ as the independent variables; adjusted R^2^ increase: 0.05–0.36, *F*_4,3052)_ = 434.99, *p* < 0.001].

### Stressor reactivity as a proxy for resilience

**Figure 4:**
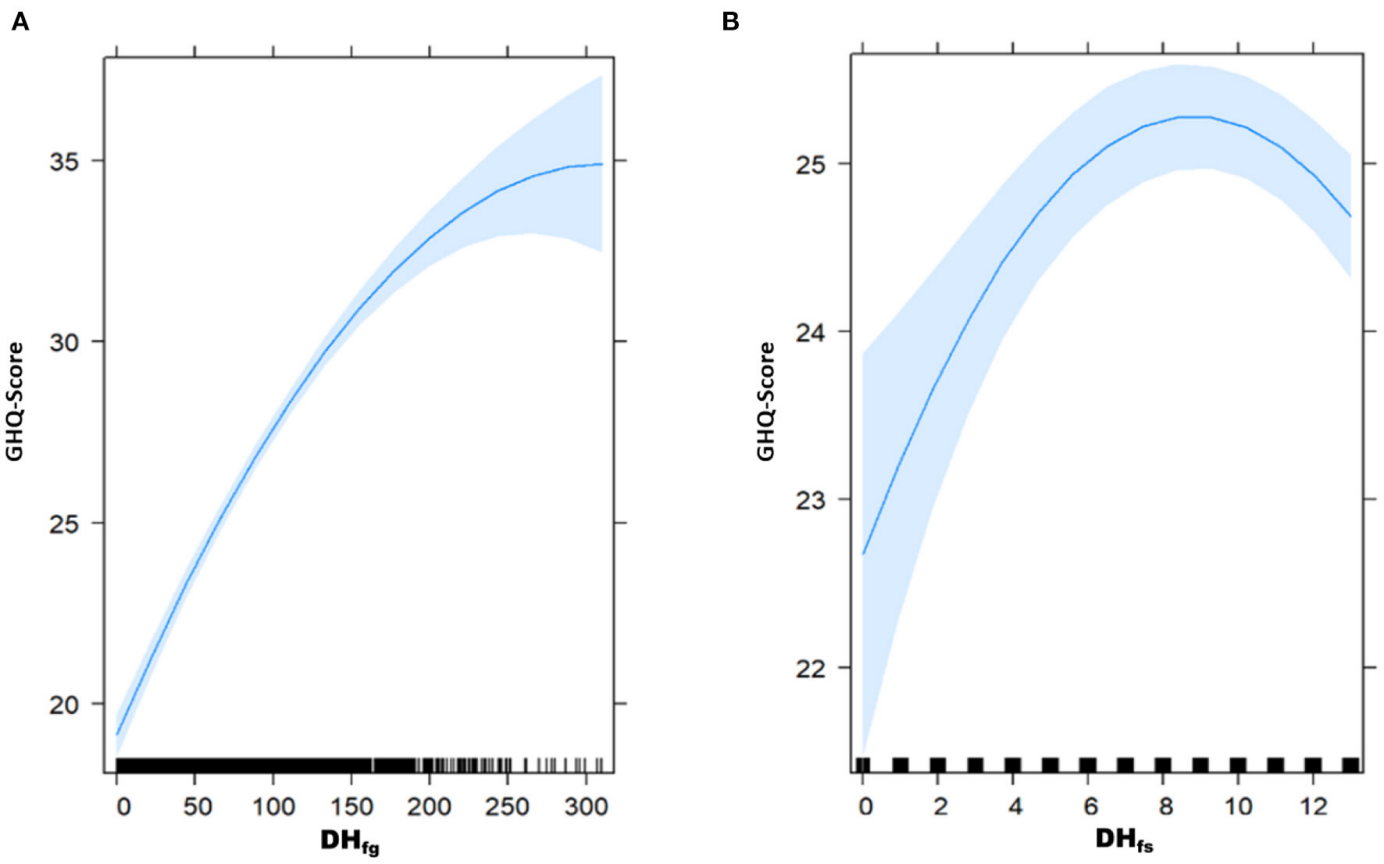
Predicted stressor-health relationship [multivariate regression analyses with GHQ score as criterion and DH_fg_ **(A)** or DH_fs_ **(B)** as predictors, respectively].

Our second goal was to estimate the SR, i.e., the stressor-mental health relationship (determined by univariate regression with GHQ score as the dependent variable and DH_fg_ and DH_fs_ as the independent variables). We found a curvilinear relationship due to a good model fit with DH_fg_ and DH_fs_ each modeled with a quadratic polynomial simultaneously (without controlling for LE, see Section Operationalization of stressor reactivity as a proxy for resilience). In the GHQ-DH regression, both predictors combined (DH_fc_) explained a substantial amount of variance of the GHQ score [adjusted R^2^ = 0.27; *F*_4,3052)_ = 277.3, *p* < 2.2e-16]. Figures 4A,B show the normative predicted stressor-mental health models with GHQ score as dependent and DH_fs_ or DH_fg_, respectively, as independent variables.

Regression analyses indicated that GHQ score is significantly related to DH_fg_ (see Figure 4A). A peak was reached at about 250 DH_fg_, and then leveled off (ß_1_= 0.079; *p* < 2e-16; ß_2_= −0.0002; *p* < 4.94e-9). The higher the DH, the worse the mental health status until a specific saturation point (see Figure 4A). Regarding DH_fs_ (see Figure 4B), regression analyses indicated that the GHQ score increased with increasing DH_fs_ and then reached a peak at about 9 DH_fs_ and flattened out. Statistically, only the main effect of the second polynomial in the GHQ- DH_fs_ score relationship became significant (ß_1_= −0.04373; *p* = 0.261; ß_2_= −0.0337636; *p* = 0.0004). The relationship between DH_fg_ and GHQ is considerably stronger than the relationship of DH_fs_ with GHQ, as visible from comparing Figure 4A with Figure 4B.

### Vulnerable groups

Our third goal was to identify risk factors resp. vulnerable groups based on the different stressors (general or corona-specific). All results are presented in Table 2. To identify general vulnerable groups, we examined the effect of sociodemographic characteristics (e.g., age) or health status (e.g., physical illness) on the DH_fg_-health relationship (controlling for DH_fs_). Gender, age, and education level significantly influenced the relationship: female gender (*p* = 0.000) and higher age (*p* = 5.11e-6) were identified as general risk factors. Participants with higher educational attainment, i.e., high school (*p* = 0.002), and those with a university degree (*p* = 0.009) were less vulnerable compared to those with low secondary or medium secondary education (also see Supplementary Figure 5). Socioeconomic status and a diagnosed mental disorder or somatic illness did not significantly influence the DH_fg_-health relationship, i.e., they were not identified as general risk factors. To identify corona-specific vulnerable groups, we examined the moderating effect of sociodemographic characteristics and health status on the DH_fs_-health relationship (controlling for DH_fg_)_._ Being diagnosed with a somatic illness significantly and negatively influenced the relationship (*p* = 0.037). Neither gender, age, SES, education level or a diagnosis of a mental disorder were identified as corona-specific risk factors.

**Table 2:** Moderation analysis—vulnerable groups (controlling for DH_fg_ or DH_fs_).

	**GHQ score x DH**_**fg**_					**GHQ score x DH**_**fs**_				
	**R^2^**	***F***	**p-value**	**b**	**p-value**	**R^2^**	***F***	**p-value**	**b**	**p-value**
**Gender**	0.285	*F*_7,3047)_ = 174.6	< 0.001	ß*_1_*= −40.74	< 0.001	0.282	*F*_7,3047)_ = 172.3	< 0.001	ß*_1_*= −9.58	0.392
				ß*_2_*= −16.60	0.150				ß*_2_*= 20.81	0.062
**Age**	0.281	*F*_10,3046)_ = 120.7	< 0.001	ß*_1_*= −1934.43	< 0.001	0.276	*F*_10,3046)_ = 174.4	< 0.001	ß*_1_*= −252.74	*0*.462
				ß*_2_*= −76.37	0.845				ß*_2_*= 492.92	*0*.138
**Education level**										
Medium secondary education	0.28	*F*_13,2992)_ = 88.41	< 0.001	ß*_1_*= 17.75	0.20751	0.270	*F*_13,86.32)_ = 2992	< 0.001	ß*_1_*= −0.62	*0*.964
				ß*_2_=* −2.82	0.84514				ß*_2_*= −3.54	*0*.794
High school				ß*_1_*= 14.08	0.405				ß*_1_*= −1.49	*0*.933
				ß*_2_*= −53.24	0.002				ß*_2_*= −2.81	*0*.869
University degree				ß*_1_*=-27.97	0.125				ß*_1_*= −0.584	*0*.974
				ß*_2_=* −49.14	0.009				ß*_2_*= 11.99	*0*.529
**SES**	0.285	*F*_7,3047)_ = 174.6	< 0.001	ß*_1_*= −2.87	0.250	0.285	*F*_7,3047)_ = 174.6	< 0.001	ß*_1_*= 2.21	*0*.418
				ß*_2_*= −0.45	0.839				ß*_2_*= 3.28	*0*.236
**Mental disorder**	0.310	*F*_7,3024)_ = 195.1	< 0.001	ß*_1_*= −28.03	0.115	0.309	*F*_7,3024)_ = 194.8	< 0.001	_1_= 0.304	0.988
				ß*_2_*= 11.83	0.436				_2_= 20.65	0.328
**Somatic illness**	0.273	*F*_7,3037)_ = 164.5	< 0.001	ß*_1_*= −5.57	0.628	0.274	*F*_7,3037)_ = 165.1	< 0.001	ß*_1_*= −23.87	0.037
				ß*_2_*= 15.86	0.169				ß*_2_*= −10.01	0.380

### Protective factors

Our fourth objective was to identify protective factors based on the different stressors (general or corona specific). To identify general protective factors, we examined the effect of resilience factors (RF; e.g., optimism) on the DH_fg_-mental health relationship (controlling for DH_fs_, age, gender, education, somatic illness, and mental disorder). All results are presented in Table 3. We found that social support is a significant moderator of the DH_fg_-health relationship (*p* = 0.047). Neither self-efficacy, LOC, nor optimism were identified as protective factors. Regarding DH_fs_-health relationship, self-efficacy (*p* = 0.004) and LOC (*p* = 0.003) were significant moderators (reinforcing effect on the second polynomial negative relationship between DH_fs_ and GHQ score). Optimism and social support were not identified as protective factors regarding coping with DH_fs_.

**Table 3:** Moderation analysis—protective factors (controlling for DH_fs_ or DH_fg_, age, gender, education, somatic illness, and mental disorder).

	**GHQ score x DH**_**fg**_					**GHQ score x DH**_**fs**_				
	**R^2^**	***F***	**p-value**	**b**	**p-value**	**R^2^**	***F***	**p-value**	**b**	**p-value**
Social support	0.343	*F*_13,2955)_ = 120.3	< 0.001	ß*_1_*= −20.56	0.084	0.342	*F*_13,2955)_ = 119.6	< 0.001	ß*_1_*= −0.004	0.100
				ß*_2_*= 20.50	0.047				ß*_2_*= −3.15	0.808
Self-efficacy	0.352	*F*_13,2955)_ = 125.1	< 0.001	ß*_1_*= −11.60	0.116	0.353	*F*_13,2955)_ = 125.6	< 0.001	ß*_1_*= −3.638	0.614
				ß*_2_*= −9.91	0.189				ß*_2_*= 20.43	0.004
LOC	0.348	*F*_13,2955)_ = 122.7	< 0.001	ß*_1_*= −5.06	0.561	0.351	*F*_13,2955)_ = 124.3	< 0.001	ß*_1_*= −14.67	0.070
				ß*_2_*= −1.89	0.822				ß*_2_*= 23.24	0.003
Optimism	0.362	*F*_13,2955)_ = 130.7	< 0.001	ß*_1_*= −8.38	0.088	0.362	*F*_13,2955)_ = 130.5	< 0.001	ß*_1_*= −2.505	0.613
				ß*_2_*= 5.01	0.255				ß*_2_*= 7.780	0.113

### In-depth analysis of previously identified vulnerable groups

Finally, moderation analyses were conducted to identify protective factors for the vulnerable groups previously identified. For the in-depth analysis, we examined the moderating effect of gender, age, education, and being diagnosed with a somatic illness on the relationship between RF (optimism, LOC, social support, and self-efficacy as independent variable) and the stressor reactivity score (SR_c_ combined predictor of DH_fg_ and DH_fs_). The results are presented in Table 4. They did not indicate any specific protective factor for the previously identified vulnerable groups (all analyses with *p* > 0.05). Rather, they showed that some protective factors lost their mitigating effect: Regarding female gender, the relationship between SR_c_ and optimism, or LOC, or self-efficacy or social support was not moderated by female gender. Neither optimism nor social support could be identified as protective factors for higher age. The otherwise positive relationship between LOC or self-efficacy and SR_c_ is even weakened by the moderating age effect (*p*_2*LOC*_ = 0.007; *p*_1*self*−*efficacy*_ = 0.0129; *p*_2*self*−*efficacy*_= 0.0001). The presence of somatic disease also weakened the otherwise positive effect of self-efficacy (*p* = 0.034) and social support (*p* = 0.046) on SR_c_. LOC and optimism were not identified as protective factors for those with a somatic illness. Neither optimism (*p* = 0.669), nor LOC (*p* = 0.193) or self-efficacy (*p* = 0.843) were identified as protective factors for lower educational attainment.

**Table 4:** Moderation analysis—in depth analysis (controlling for age, gender, education, somatic illness, and mental disorder, resp.).

	**SR**_**c**_ **x female gender**					**SR**_**c**_ **x higher age**					**SR**_**c**_ **x somatic illness**				
	**R^2^**	**F**	**p-value**	**b**	**p-value**	**R^2^**	**F**	**p-value**	**b**	**p-value**	**R^2^**	**F**	**p-value**	**b**	**p-value**
Social support	0.098	*F*_10,2958)_ = 33.17	< 0.001	ß = 0.037	0.292	0.098	*F*_11,2957)_ = 30.31	< 0.001	ß*_1_*= −0.45	0.645	0.099	*F*_10,2958)_ = 33.49	< 0.001	ß = −0.07	0.046
									ß*_2_*= −1.33	0.123					
Self-efficacy	0.108	*F*_10,2958)_ = 36.88	< 0.001	ß = 0.056	0.110	0.107	*F*_11,2957)_ = 35	< 0.001	ß*_1_*= 1.44	0.013	0.108	*F*_10,2958)_ = 37.01	< 0.001	ß = −0.08	0.034
									ß*_2_*= −3.50	0.000					
LOC	0.100	*F*_10,2958)_ = 34.02	< 0.001	ß = 0.006	0.872	0.100	*F*_11,2957)_ = 31.67	< 0.001	ß*_1_*= −0.20	0.838	*0*.100	*F*_10,2958)_ = 34.02	< 0.001	ß = −0.00	0.927
									ß*_2_*= −2.55	0.007					
Optimism	0.117	*F*_10,2958)_ = 40.17	< 0.001	ß = 0.024	0.488	0.116	*F*_11,2957)_ = 36.5	< 0.001	ß*_1_*= 0.33	0.745	0.117	*F*_10,2958)_ = 40.19	< 0.001	ß = 0.03	0.415
									ß*_2_*= −0.89	0.354					

## Discussion

The present study gained deeper insight into the mental health burden and its contributing general and corona-specific stressor load, potential risk and protective factors as well vulnerable groups during an early stage of the SARS-CoV-2 pandemic in a representative sample of the German adult population. At first, we analyzed the stressor load of the representative sample regarding frequency and burden. 83–60% were affected by the general DH_fg_ such as housekeeping, bad news in media, negative political incident, own physical complaints or bad weather. Their average perceived burden, however, was relatively low (even almost not burdensome, ranging from 1.23 to 1.71). The most burdensome DH_bg_ were sleeping problems and own physical complaints (affecting 51–57% of respondents), bad behavior of others and time pressure (38%), as well as financial problems (28%). Among corona-specific DH_fs_, almost all the responds (97–84%) were affected by media reports, loss of ability to participate in recreational activities or in important social events, not feasible private or professional travels, loss of social contacts, and less physical activity than usual. The perceived stressor burden had a high influence on mental health outcome (GHQ), i.e., higher occurrence of DH, resulted in higher mental burden. It explained about one-third of the variance: 36% by combined stressors, 31% by DH_bg_, and 24% by DH_bs_ alone, respectively. In comparison, almost 10% less variance of mental health is explained, if instead of stressor burden, their frequency of occurrence (DH_fg_ and DH_fs_) is used (R^2^ adjusted: 26.56). Our third and fourth goal were to identify general resp. corona-specific vulnerable groups and protective factors: female gender, higher age, and lower education level (low secondary or medium secondary education) were identified as general, somatic diseases as a corona-specific risk factor. Whereas self-efficacy and locus of control (LOC) proved to be corona-specific protective factors, social support was not: a high degree of social support attenuated mental health among high occurrence of DH_fs_. Further analysis did not indicate any specific protective factor for the previously identified vulnerable groups, they even showed that older age and being diagnosed with a somatic illness had a negative impact on RF, in the sense of attenuating the positive influence of LOC, self-efficacy, and social support on stressor reactivity (SR). In the following we will discuss the results in more detail.

### Stressor load

An impact of the crisis on participants' mental health was evident since our sample showed a higher mean GHQ score (12.49, SD = 6.58) compared to pre-pandemic mean scores (mean = 9.70, SD = 4.94) measured in a representative German sample in 2012 (64), but also lower scores compared to the most intense phase of the lockdown in Europe (March 22 to April 19, 2020) (mean = 15.5, SD = 6.2) measured in Europe (29). This is in line with the observation that the impact of COVID-19 on mental health varied due to different time points of examination, different restrictions in different countries (6, 65), lockdown situations (66, 67) or during isolation in suspected COVID-19 cases (7, 19, 68). The German population was relatively less affected in an international comparison (i.e., less fear of job loss or financial losses due to government intervention) (29), but nevertheless showed elevated generalized or COVID-19-related anxiety symptoms as well as depressive symptoms (20, 69) and psychological distress compared with pre-pandemic data (34). Consistent with previous findings in a representative German sample (52) housekeeping, time pressure, and bad weather were also the most frequently occurring DH_g_ in pre-pandemic times. However, in our sample, we observed a shift in attention to Corona-related issues, which was also identified from Veer et al. (29): while pre-pandemic bullying, problems with a pet and conflicts or disagreements with relatives were identified as the most distressing DH_g_ (52), in our study negative political events (DH_g_) were also mentioned frequently. This is not only consistent with other findings during the pandemic in Germany (29), but also known to be a risk factor for mental burden (7, 20). The shift in attention to Corona-related issues could also be influenced by the use of fear-based media coverage implemented to prompt people to strictly adhere to the established guidelines (14, 15). A meta-analysis by Witte and Allen (70) showed that the dissemination of fear appeals, which are regularly used in other contexts (cf. deterrent images of a black lung in smoking prevention), can lead to behavioral changes when the concerned person feels able to deal with the threat. A lack of expectation to be able to deal with it, however, can lead to defensive reactions (e.g., questioning the meaningfulness of the measures). A paradoxical societal effect regarding the fear-based media coverage would be possible: The more mentally burdened the population, the lower the expectation of self-efficacy in dealing with the threat and therefore compliance with individual health-protective behaviors decreases (34). This might result in higher infection rates and, as a consequence, in even more fear-based appeals (71). The occurrence frequency of Corona-related stressors (e.g., corona media reports or negative political events) was particularly notable in our study (80.73 and 72.42%, respectively). At the same time, no excessive burden to Corona-related stressors was found in our study. One explanation could be the decrease in reports of deaths, as the mortality rate was often perceived as particularly threatening (72). At the time of our survey in Germany the mortality rate was just under 5% (40, 73). Nevertheless, worldwide corona reports were mostly negative (74) and therefore affected behavior (e.g., social distancing, lower willingness to be vaccinated) and emotions (e.g., loneliness) (72). The associated aversive emotions may also have led to psychological defense mechanisms: As a link between psychological distress and higher media consumption times has also been demonstrated during the pandemic (74, 75), experts recommend curbing media consumption so that negative news are not permanently consumed (76). Compared with the findings of Veer et al. (29), who cited serious consequences (such as death or hospitalization of a loved one and concern about one's infection) as the primary corona stressor, concerns in our study shifted toward financial, health-related (e.g., sleep problems, injury or illness, less physical activity than usual, corona reporting), and leisure problems (non-participation in social events, fewer social contacts, neither personal nor professional travel) during the phase investigated in this study.

### Resilience and mental health

The mean scores of self-estimated resilience of our respondents (BRS: mean = 3.31; SD = 0.78) was similar to pre-pandemic data [mean = 3.35; SD = 0.95, for a study with *N* = 1,128 respondents in Germany (77)], but marginally lower as compared to other results obtained during the pandemic [April 2020, mean = 3.41; SD = 0.49, German sample sizes: 1.012 (3)]. However, these resilience scores are self-estimates of probands who are asked to describe how quickly they bounce back from stressful events, but no “real life” measures how their mental health relates to the number and burden of stressful experiences. We therefore calculated the SR score as a proxy for resilience as current psychological responsiveness (as measured by the GHQ) to daily stressors [as measured by MIMIS and/or a DynaCORE item selection (29)]. In our cross-sectional data, we observed a concave DH_fg_-health relationship (see Figure 4): The leveling of the mental health-stressor relationship, i.e., that it flattens out from a certain number of daily hassles, shows that in our sample from around 250 DH_fg_ onward, further stressors have a less severely deteriorating effect on mental health. This could carefully be interpreted as a possible adaptation process to stressors (78). Our finding that corona-specific stressors frequently occurred, but were not perceived as burdensome by a large majority, may also be interpreted as hint for a possible adaptation process. Manchia et al. (33) showed that, after the restrictions were lifted, a large portion of the population recovered from the pandemic related stressor impact. This could be attributable to the corona-induced slowdown (79), but also to a successful adaption process described as “psychological gain from adversity” as suggested by Ahrens et al. (80). The sole occurrence of corona-specific stressors had little impact on mental health (corona-specific stress items clarified only 5% of the variance of the GHQ score additionally and non-significant effect in the regression; first polynomial, i.e., no decreasing GHQ score with increasing DH_fs_), which implies that DH_g_ had more impact on the mental health than DH_s_. Counterintuitive our results also show with a higher incidence of corona-specific DH less mental burden (significant effect in the second polynomial). This might be a result of the summed binary operationalization of corona-specific DH_fs_ (left skewed distribution), i.e., just under half of the respondents reported an occurrence corresponding to data point 13 (see Figure 4B) of the DH_fs_, leading to an underrepresentation of data points to the left of it.

The combined stressors (general and corona-specific) explained more variance of the GHQ score than the predictors separately. Furthermore, the perceived stressor burden is a better predictor (36%) than the frequency of its occurrence (26%), which is in line with other findings (29, 81) as well as with the Transactional Model of Stress and Coping (28), highlighting that the stress reaction depends on the specific appraisal of the stressor (i.e., as harm/loss, threats, and challenge) in relation to the resources available.

### Vulnerable groups, protective factors, and in-depth analysis of previously identified vulnerable groups

In line with other findings (2, 3, 9, 11, 19–23), women were identified as particularly vulnerable to psychological stress during the pandemic. Women's BRS (mean = 3.22, SD = 0.74) and GHQ scores (mean = 13.25; SD = 6.81) in our sample showed lower resilience and mental health, respectively, than men's BRS score (mean = 3.41, SD = 0.74) or GHQ score (mean = 11.71; SD = 6.23), which is consistent with other findings during the pandemic (8, 80). When considering the impact of general or corona-specific stressors on mental health, female gender was found to be a general risk factor. This may indicate that it is not a corona-specific vulnerability, e.g., because of domestic childcare, as has been cited in previous research (7, 33, 82). We also found lower education level (low and medium secondary education level) as risk factor: the GHQ score for the lower education groups continues to increase with more occurrence of DH_fg_. However, this result is based on comparatively few data points, i.e., few respondents had more than 250 DH_fg_ (shown by the dashes on the x-axis in Supplementary Figure 5), so it should be interpreted with caution. At the same time, this result is consistent with other studies (8, 17, 83).

In many studies, younger age groups ( ≤ 40 years) are highlighted as particularly vulnerable during crisis: Older people are suggested to be protected through life experiences, thus more problem-solving skills and a stronger locus of control, and ultimately a more efficient psychological coping and adaptive capacity during COVID-19 (2, 7, 22, 30). In our study, this finding could not be replicated, which may be due to our use of SR as a proxy of resilience (predicting mental health as a function of stress). Furthermore, although older people tend to be exposed to fewer stressors (19, 36, 84), they might be likely to be more responsive to them. We identified a diagnosed somatic disease as a risk factor for corona-specific stressors. Somatic diseases are a well-researched risk factor for mental health (7, 9, 17) and the risk for somatic diseases and infections grows with increasing age (31). As Taquet et al. (68) showed in a retrospective US cohort study in August 2020, there is also a bidirectional association between SARS-CoV-2 infection and risk of mental disorder and vice versa. Regarding resilience factors as protective factors for mental burden, self-efficacy and LOC were found to be corona-specific protective factors, which is consistent with earlier findings (7, 30, 33). However, contrary to our expectations (2, 3, 29, 69), optimism and social support were not found to be protective factors in our study. Individuals reporting high levels of social support were even more affected by stressors on GHQ. This implies that this resilience factor loses its effect in times of social distancing. This would at least be supported by the study results that reported increased loneliness (6, 85). We could not demonstrate a specific protective factor for the previously identified vulnerable groups, i.e., females, older age, and lower education level. In contrast, the elderly and the somatically ill showed lower RF scores, which otherwise has a positive effect on resilience (LOC, self-efficacy, and social support) (24, 25). This could be seen in the context of the findings of Fritz et al. (27) on the interaction of different protective factors: Protective factors influence each other (intensification or inhibition), and network connectivity between protective factors is less responsive in vulnerable people, making already vulnerable groups even more vulnerable to stress. This may explain why vulnerable groups, e.g., elderly, or somatic ill, but also known from other findings have shown to be more vulnerable to the adversities of the pandemic (8, 68, 86).

Our data provide insights in especially vulnerable groups (women, older age, and lower education level) and specific starting points in order to strengthen protective factors, by identifying self-efficacy and LOC as protective factors during the pandemic. However, since we only researched a limited number of resilience factors, there are certainly other protective factors which we did not study here but which might be important to fostering mental health (e.g., certain coping styles). In other peri-pandemic analyses, for example, positive appraisal style (29, 80) was identified as a protective factor.

### Strengths and limitations

The strengths of the study are the large and representative sample, the use of well-established standardized measures that allow comparisons with other pre- as well as pandemic populations. We also not only relied on self-estimated resilience capability of the probands, but used the SR score (62) to describe a proxy for resilience which relates mental burden to the perceived stressor load. The study is also new as it disentangles the relative contributions of general and corona-specific DH to mental burden and resilience.

On the other hand, our study has the following limitations: First, we collected the representative data in an online survey, so we cannot exclude selection bias. In addition, we did not collect longitudinal data, which are considered the gold standard (52, 62). Furthermore, changes in mental health were assessed retrospectively over the past 2 weeks, which might have led to memory bias. In addition, the survey was conducted during a less severe phase of the pandemic, meaning that some of the restrictions (e.g., social distancing, closing of restaurants, closing of recreational and cultural facilities) had already been lifted. Although the corona pandemic caused measurable burden, it is likely that at a different time point during the course of the pandemic, corona-specific stress would have been more pronounced. Due to a reasonable questionnaire length, we had to severely limit the constructs we examined, which is why we only queried a selection of the RF and only examined a selection of corona-specific stressors. The last made the comparison to the general stressors more difficult. To the extent that resilience was operationalized as an outcome (stressor exposure relative to mental health), survey inaccuracies may have crept in: It cannot be ruled out that mental health problems have their genesis in other stressors or LE that were not surveyed in this instrument.

### Conclusions

The corona pandemic seems to have an impact on the general population in the sense that corona-specific stressors were perceived but not as burdensome during this phase of the pandemic (e.g., general stressors such as bad news in the media or own physical complaints). At the same time, the corona-specific stressor load was hardly perceived as a burden: This is also reflected in the low impact on mental health. This result should be viewed in the context of the data collection period, for which some restrictions had already been lifted and may have led to adaption. As in many other studies, we were also able to show that there are vulnerable groups (women, lower education level, older age, and somatic illness), who are at a higher risk of being negatively affected by the pandemic. In addition, specific protective factors (self-efficacy and LOC) for the corona-specific stressors were identified in our study. Extending previous studies, we were also able to show that older people and people with somatic illnesses are particularly affected by corona-specific stressors, which again underlines their need for special support regarding an adaptive coping during and after the pandemic.

## Data availability statement

The raw data supporting the conclusions of this article will be made available by the authors, without undue reservation.

## Ethics statement

The studies involving human participants were reviewed and approved by Ethics Committee of the Department of Psychology and Sports Science of the Johann-Wolfgang-Goethe University Frankfurt (Prof. Andreas Klein). The patients/participants provided their written informed consent to participate in this study.

## Author contributions

LH, IH, DG, and SF: conceptualization, methodology, and validation. GK: statistical analysis and data visualization. LH: investigation and writing of the original draft. KL and RD: project management, monitoring, and validation. IH, DG, and KL: writing—revised draft, monitoring, and validation. All authors contributed to the article and approved the submitted version.

## Funding

This work was supported by the Leibniz-Gemeinschaft (Grant number: K83/2017 Resilience Factors in a Diachronic and Intercultural Perspective) to KL and a research grant from the Johannes Gutenberg University Mainz Top-level Research Area 40,000 Years of Human Challenges: Perception, Conceptualization and Coping in Premodern Societies funded by Research-Initiative Rhineland-Palatinate (2019–2023), coordinated by A. Busch and H. Frielinghaus, to KL and RD.

## Conflict of interest

The authors declare that the research was conducted in the absence of any commercial or financial relationships that could be construed as a potential conflict of interest.

## Publisher's note

All claims expressed in this article are solely those of the authors and do not necessarily represent those of their affiliated organizations, or those of the publisher, the editors and the reviewers. Any product that may be evaluated in this article, or claim that may be made by its manufacturer, is not guaranteed or endorsed by the publisher.
