# Supplementary material for: Effects of general and corona-specific stressors on mental burden during the SARS-CoV-2 pandemic in Germany
Source: Front Public Health. 2022 Nov 22;10:991292. doi: 10.3389/fpubh.2022.991292 (PMC9724653; doi:10.3389/fpubh.2022.991292)
Supplement: Supplementary file 1 [file Data_Sheet_1.PDF]

Supplementary Material

1 Survey questionnaire

Survey questionnaire	Description	Number of items	Time period	Response format
Sociodemographics (Part 1)	General (age, gender, …)<br>Origin and language<br>Social situation<br>Occupational situation	3 items<br>7 items<br>2 items<br>10 items	-	Selection | Open response format<br>Yes | No, selection<br>Selection<br>Yes | No, selection
Corona Pandemic, a selection of 13 items of the international survey DynaCORE (Veer et al., 2021)	Corona-specific Daily Hassles (DH_s)<br><br>Frequency (DH_{fs}) and burden (DH_{bs})	13 items	Up to the last 7 days	Selection | from 'not at all burdensome' to 'very burdensome' on a five-point Likert scale; did not occur
Pro-diversity beliefs (Kauff & Wagner, 2012)	Cultural diversity	2 items	-	1 'not at all' | 2 'rather not' | 3 'rather' | 4 'fully'
Individual reflective values (Individuelle reflexive Werte), (Hermann, 2004)	Values	16 items	-	'not very important' to 'very important' on a seven-point Likert scale
Political attitudes (Sozio-Politische Einstellungen) (Fischer & Kohr, 2014)	Non-political attitude/ political engagement	16 items	-	'correct' | 'not correct'
Social Identity (Doosje, Ellemers, & Spears, 1995)	Social identification	4 items	-	1 'do not agree at all' | 2 'tend to disagree' | 3 'neither' | 4 'tend to agree' | 5 'agree completely'
General Health Questionnaire (GHQ-12) (Goldberg et al., 1997)	Mental health	12 items	The last few weeks	1 'no, not at all'/ 'better than usual'/ 'more than usual' | 2 'not worse than usual'/ 'not more than usual'/ 'as usual' | 3 'worse than usual'/ 'more than usual'/ 'heavier than usual'/ 'less than usual' | 4 'much worse than usual'/ 'much more than usual'/ 'much heavier than usual'/ 'much less than usual'

Brief Resilience Scale (BRS)<br>(Chmitorz et al., 2018)	Ability to bounce back quickly from stress	6 items	-	1 'do not agree at all' | 2 'tend to disagree' | 3 'neither' | 4 'tend to agree' | 5 'agree completely'
Perceived Stress Scale (PSS-4)<br>(Cohen, Kamarck, & Mermelstein, 1983)	Subjective perceived stress	4 items	Within last month	1 'never' | 2 'rarely' | 3 'sometimes' | 4 'frequently' | 5 'very often'
Mainz Inventory of Microstressors (MIMIS)<br>(Chmitorz et al., 2020)	general Daily Hassles (DH_g)<br><br>Frequency (DH_{fg}) and burden (DH_{bg})	58 items	Up to the last 7 days	1-7 days, did not occur | from 'not at all burdensome' to 'very burdensome' on a five-point Likert scale
Life Event	Stressful event	1 item	Within the last 12 months	Open response format
Response To Stressful Experiences-Scale (RSES-4)<br>(Ponder, Prosek, & Sherrill, 2021)	Evaluation of the stressful event	4 items	-	1 'do not agree at all' | 2 'tend to disagree' | 3 'neither' | 4 'tend to agree' | 5 'agree completely'
Optimism-pessimism (SOP-2)<br>(Kemper, Beierlein, Kovaleva, & Rammstedt, 2012)	optimism-pessimism	2 items	-	from 'not optimistic/ pessimistic at all' to 'very optimistic/ pessimistic ' on a seven-point Likert scale
Locus of control (IE-4)<br>(Kovaleva, Beierlein, Kemper, & Rammstedt, 2012)	internal and external locus of control	4 items	-	1 'does not apply at all' | 2 'applies a little' | 3 'applies somewhat' | 4 'applies fairly' | 5 'applies fully'
Self-effiacy (ASKU)<br>(Beierlein, Kovaleva, Kemper, & Rammstedt, 2012)	self-effiacy	3 items	-	1 'does not apply at all' | 2 'applies a little' | 3 'applies somewhat' | 4 'applies fairly' | 5 'applies fully'
Social support (OSSS-3)<br>(Kocalevent et al., 2018)	Perceived social support	3 items	-	response format differs on a 4 resp. 5-point Likert scale
Sociodemographics<br>*(Part 2)*	Health<br>Alcohol consumption<br>Tobacco consumption<br>Consumption of illegal drugs	4 items<br>2 items<br>3 items<br>3 items		Yes | No, Open response format<br>Selection<br>Yes | No, selection<br>Yes | No, selection
				**182 items**

Figure 5

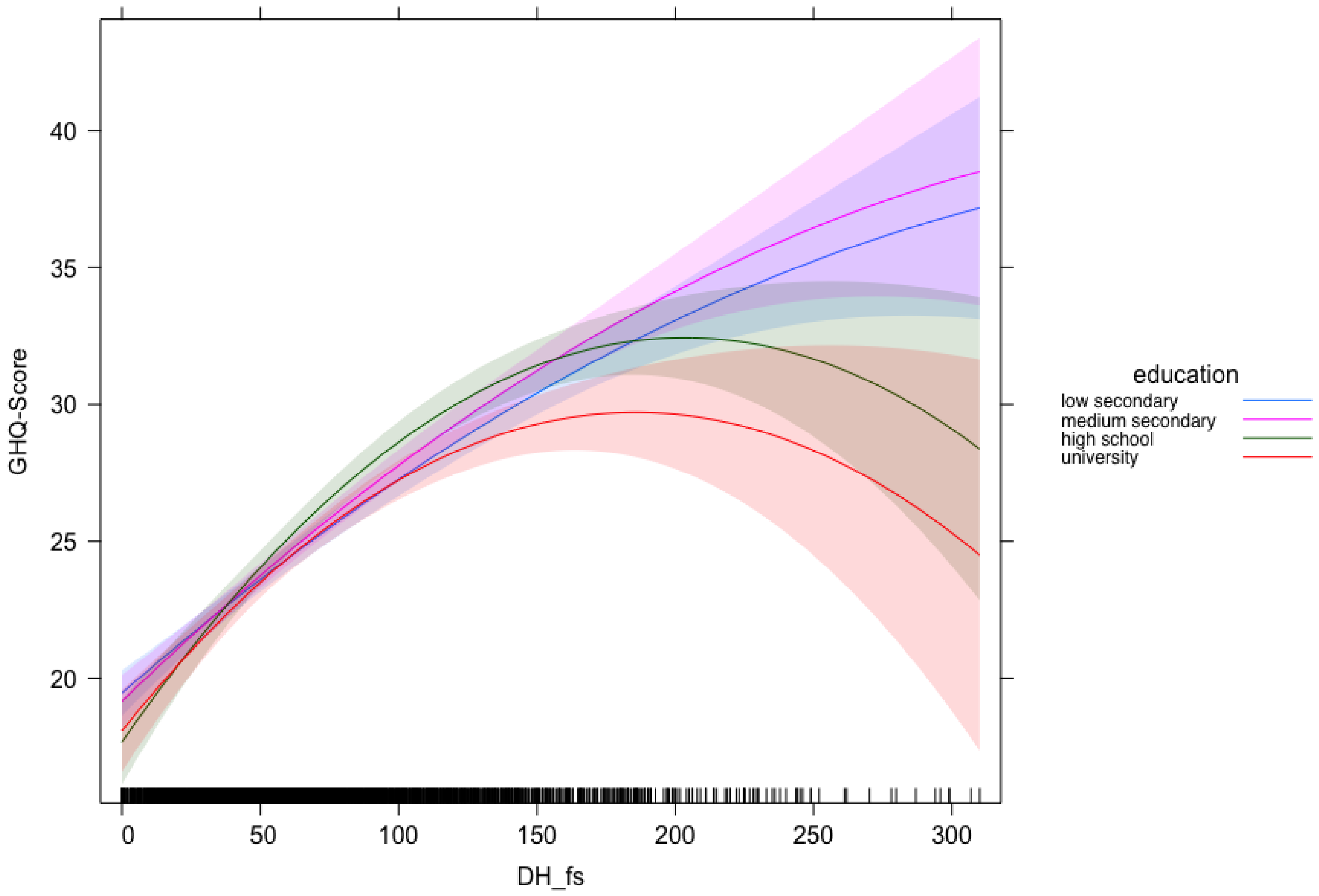

Figure 5. Influence of education level on DH_{fs}-GHQ-Relationship (multivariate regression analyses with GHQ score as criterion and DH_{fs} as predictors, and education as moderator).

3 Literature

Beierlein, C., Kovaleva, A., Kemper, C. J., & Rammstedt, B. (2012). ASKU-Allgemeine Selbstwirksamkeit Kurzskala.

Chmitorz, A., Kurth, K., Mey, L., Wenzel, M., Lieb, K., Tüscher, O., Kubiak, T., & Kalisch, R. (2020). Assessment of microstressors in adults: Questionnaire development and ecological validation of the mainz inventory of microstressors. *JMIR mental health, 7*(2), e14566. doi: 10.2196/14566

Chmitorz, A., Wenzel, M., Stieglitz, R., Kunzler, A., Bagusat, C., Helmreich, I., Gerlicher, A., Kampa, M., Kubiak, T., & Kalisch, R. (2018). Population-based validation of a German version of the Brief Resilience Scale. *PloS one, 13*(2), e0192761. doi: 10.1371/journal.pone.0192761

Cohen, S., Kamarck, T., & Mermelstein, R. (1983). Perceived stress scale (PSS). *J Health Soc Beh, 24*, 285.

Doosje, B., Ellemers, N., & Spears, R. (1995). Perceived intragroup variability as a function of group status and identification. *Journal of experimental social psychology, 31*(5), 410-436. doi: 10.1006/jesp.1995.1018

Fischer, A., & Kohr, H. (2014). Sozio-Politische Einstellungen: GESIS.

Goldberg, D. P., Gater, R., Sartorius, N., Ustun, T. B., Piccinelli, M., Gureje, O., & Rutter, C. (1997). The validity of two versions of the GHQ in the WHO study of mental illness in general health care. *Psychological medicine, 27*(1), 191-197.

Hermann, D. (2004). Individuelle reflexive Werte Retrieved 27.05.2022, 2022, from https://doi.org/10.6102/zis135

Kauff, M., & Wagner, U. (2012). Valuable therefore not threatening: The influence of diversity beliefs on discrimination against immigrants. *Social Psychological and Personality Science, 3*(6), 714-721. doi: 10.1177/1948550611435942

Kemper, C. J., Beierlein, C., Kovaleva, A., & Rammstedt, B. (2012). Eine Kurzskala zur Messung von Optimismus-Pessimismus: GESIS Working Paper 15.

Kocalevent, R.-D., Berg, L., Beutel, M. E., Hinz, A., Zenger, M., Härter, M., Nater, U., & Brähler, E. (2018). Social support in the general population: standardization of the Oslo social support scale (OSSS-3). *BMC psychology, 6*(1), 1-8.

Kovaleva, A., Beierlein, C., Kemper, C., & Rammstedt, B. (2012). Eine Vier-Item-Skala zur Erfassung von internaler und externaler Kontrollüberzeugung (IE-4): GESIS.

Ponder, W. N., Prosek, E. A., & Sherrill, T. (2021). Validation of the Adapted Response to Stressful Experiences Scale (RSES-4) Among First Responders. *Professional Counselor, 11*(3), 300-312.

Veer, I. M., Riepenhausen, A., Zerban, M., Wackerhagen, C., Puhlmann, L. M., Engen, H., Köber, G., Bögemann, S. A., Weermeijer, J., & Uściłko, A. (2021). Psycho-social factors associated with mental resilience in the Corona lockdown. *Translational psychiatry, 11*(1), 1-11.
